# The use of CAM and conventional treatments among primary care consulters with chronic musculoskeletal pain

**DOI:** 10.1186/1471-2296-8-26

**Published:** 2007-05-04

**Authors:** Majid Artus, Peter Croft, Martyn Lewis

**Affiliations:** 1 Primary Care Musculoskeletal Research Centre, Keele University, Stoke on Trent, Staffordshire, ST5 5BG, UK

## Abstract

**Background:**

Chronic musculoskeletal pain is the single most cited reason for use of complementary and alternative medicine (CAM). Primary care is the most frequent conventional medical service used by patients with pain in the UK. We are unaware, however, of a direct evidence of the extent of CAM use by primary care patients, and how successful they perceive it to be.

**Methods:**

**Aims and objectives:**

To determine CAM use among patients with chronic musculoskeletal pain who have consulted about their pain in primary care.

**Study design:**

Face-to-face interview-based survey.

**Setting:**

Three general practices in North Staffordshire.

**Participants:**

Respondents to a population pain survey who had reported having  musculoskeletal pain in the survey and who had consulted about their pain  in primary care in the previous 12 months as well as consenting to further  research and agreeing to an interview. Information was gathered about  their pain and the use of all treatments for pain, including CAM, in the  previous year.

**Results:**

138 interviews were completed. 116 participants (84%) had used at  least one CAM treatment for pain in the previous year. 65% were current  users of CAM. The ratio of over-the-counter CAM use to care from a CAM  provider was 3:2. 111 participants (80%) had used conventional treatment.  95 (69%) were using a combination of CAM and conventional treatment.  Glucosamine and fish oil were the most commonly used CAM treatments (38%,  35% respectively). Most CAM treatments were scored on average as being  helpful, and users indicated that they intended to use again 87% of the  CAM treatments they had already used.

**Conclusion:**

We provide direct evidence that most primary care consulters with  chronic musculoskeletal pain have used CAM in the previous year, usually  in combination with conventional treatments. The high prevalence and wide  range of users experiences of benefit and harm from CAM strengthen the  argument for more research into this type of medicine to quantify benefit  and assess safety. The observation that most users of conventional  medicine also used CAM suggests a continuing need for more investigation  of effective pain management in primary care.

## Background

Surveys have suggested that use of complementary and alternative medicines (CAM) is high and increasing worldwide [1]. Longitudinal studies in the UK between 1993 [2] and 1999 [3], 1995 [4] and 2001 [5] and between 1998 [6] and 2006 [7] and in the USA between 1990 [8] and 1997 [9] have confirmed the trend. CAM is most commonly used for chronic pain and in particular musculoskeletal pain [7] and is often used in combination with conventional therapies [10]. GPs are the conventional medical practitioners most frequently consulted for chronic pain in the UK [11].

A number of surveys have been conducted in the UK to explore CAM use. Some of these were general population surveys [2,3,11-13], surveys among patients with definitive rheumatologic diagnoses attending hospital clinics [14-18], or surveys of healthcare professionals exploring their patients' use of CAM and access to it [19,4,5,23].

In primary care in the UK, surveys targeted healthcare professionals rather than patients. [4,5] We are not aware of surveys conducted in the UK that have directly explored CAM use among primary care patients who suffer from chronic musculoskeletal pain. Evidence suggests that users do not necessarily access CAM through primary care and also they are often reluctant to inform their doctors of their use of these treatments [9,24]. Figures on access to CAM through primary care and on health professionals' provision of CAM, therefore, might have under-represented actual CAM use among primary care patients.

**Figure 1:**
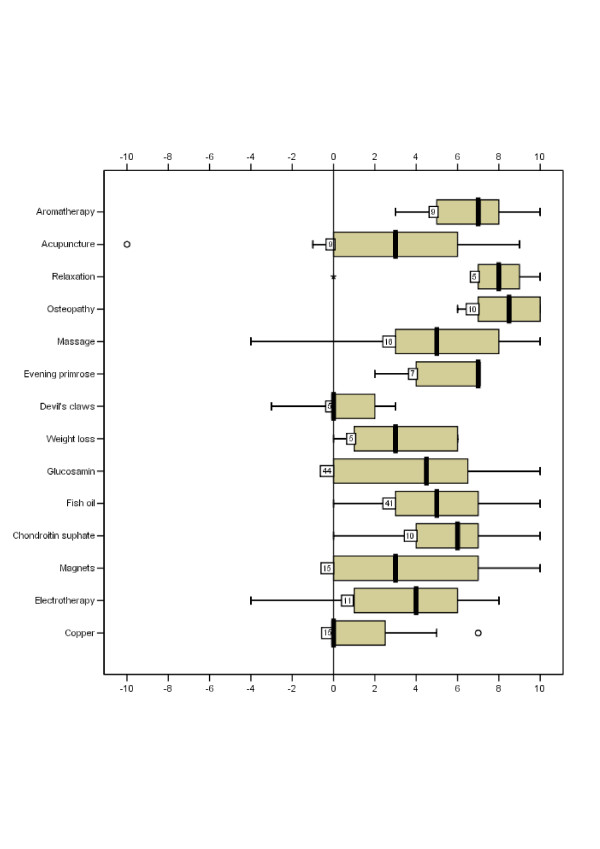
Boxplot summary of perceived helpfulness scores on the VAS for the most commonly used CAM treatments. Values represented are number of users and 5-number summary (median, 25th and 75th percentiles and range) and outlier observations (data that lie outside the interval: median ± 1.5× mid-spread).

We are not aware of previous surveys of primary care patients that have enquired about the perceived helpfulness of treatments from the patients' perspectives. This is important for understanding healthcare seeking behaviour among chronic pain sufferers, for informing effective pain management in primary care and because of potential safety issues related to CAM use.

We wanted to address these issues with particular focus on musculoskeletal pain sufferers who are using primary care in the UK. We have therefore investigated the pattern of CAM use in a sample of chronic musculoskeletal pain patients who were consulting primary care in the UK. Our hypothesis was that this group of patients would have a higher prevalence of CAM use than general population samples or pain sufferers generally. This was based on the idea that this group will have selectively more severe problems than the general population because they have sought health care and because chronic musculoskeletal pain is often unresponsive to conventional primary care treatments

## Methods

### Setting and study population

The population consisted of adults, aged 18 years and older, registered with three general practices in the North Staffordshire General Practice Research Network.

The sample was drawn from responders to previous postal health surveys [25,26] conducted in the Network. They had agreed to further contact and to use of their medical records for specific research purposes. Approval for this specific study was obtained from the North Staffordshire Ethics Committee. We first identified all patients who had reported pain in the surveys and who had consulted their general practitioners during a defined 12-month period with any condition or syndrome of chronic musculoskeletal pain as identified from their computerised medical records. The quality of the coded consultation data in the Network practices is audited regularly and this has been reported previously in the literature [27]. The main inclusion criterion was a record of a consultation for musculoskeletal pain during the 12 months prior to the interview. We excluded patients with pain caused by or associated with malignancy (primary or secondary), visceral (gynaecological or general surgical), vascular, neuropathic conditions or infections. We also excluded individuals who were known by their GPs to have impaired cognitive function. Language was not an excluding criterion. GPs reviewed the final list to further exclude patients on the basis of terminal illness or inappropriateness for social reasons.

### Sample size

Anticipating the prevalence of CAM use on the basis of previous surveys to be at least 30%-50%, we calculated that 180 patients would need to be recruited in order to detect at least 20% difference in prevalence between different socio-demographic groups with 80% power. Given the need for an in-depth interview, we predicted a response rate of 40–50%. The total number of potential participants identified was 427. We invited all of them to participate in order to generate our calculated sample size.

### Design

The methods used were a combination of a face-to-face structured interview, a self-completed questionnaire and data collection from medical records. The interview questionnaire consisted of three sections. Section A was about the timing of pain and its location. Section B was about treatments used for pain control and consisted of eight questions. Section C included questions about socio-demographic characteristics. For social and occupational classifications we used the Office for National Statistics socio-economic classification (NS – SEC) [28].

Section B started with an open question about treatments used for pain. A list of treatment names was not used at this stage and it was clearly explained to participants that we wanted to know of anything they had used or anything done to help them with their pain. After answers were obtained to that question, a list of treatments was then introduced (Table 1) and participants were asked to answer the same question, again, this time with the aid of the list. The list included all types of treatments (conventional and items of self-care as well as CAM) which could be used for pain, regardless of type, classification, definition, local availability, prevalence of use or effectiveness.

**Table 1:** A list of treatments addressed in the study.

Treatments included in interview list			
CAM		Conventional	Additional CAM treatments reported by participants

1.Aromatherapy	26.Muscle energy techniques	1.Ultrasound	1.Exercises
2.Homeopathy	27.Therapeutic touch	2.Vitamins	2.Heat therapy
3.Magnets	28.Alexander Technique	3.Joint injections	3.Heat cream
4.Copper bracelets	29.Massage Therapy	4.Occupational Therapy	4.Biocomfort
5.Evening primrose	30.Phytodolor	5.Operations	5.Dog oil
6.Reflexology	31.Osteopathic manipulation	6.Podiatry	6.Glyco-nutrient
7.Naturopathy	32.Electrotherapy	7.Psychotherapy	7.Honey & vinegar
8.Feverfew extract	33.Chondroitin sulphate	8.Aspirin	8.Cold pack
9.Blackcurrant seed oil	34.Glucosamin	9.Celecoxib	9.Geranium & eucalyptus
10.Ayurvedic herbs	35.Lifestyle program	10.Co-codamol	10.Reiki
11.Borage seed oil	36.Minerals	11.Co-codaprin	11.Swimming
12.Devil's claw	37. Weight loss program	12.Co-dydramol	12.Dowsing
13.Ginger	38.Active release technique	13.Co-proxamol	13.Electrical massage
14.Thunder God Root	39.Myofascial release	14.Diclofenac sodium	14.Florid acid
15.Acupuncture	40.Soft tissue mobilization	15.Ibuprofen	15.Lavender oil
16.Acupressure	41.Biofeedback	16.Indomethacin	16.Singapore balm
17.Tumaric	42.Guided imagery	17.Mefenamic acid	
18.Hypnosis	43.Pilates	18.Meloxicam	
19.Energy Healing	44.Prayer	19.Naproxen	
20.Fish oil	45.Relaxation	20.Nefopam	
21.Willow bark extract	46.Tai Chi	21.Paracetamol	
22.Meditation	47.Yoga	22.Piroxicam	
23.Pet Therapy	48.Hydrotherapy	23.Rofecoxib	
24.Chiropractic	49.Chelation		
25.Craniosacral Therapy	50.Serums		
	51.Vaccines		

The interviewer proceeded by asking detailed questions as follows about each of the treatments used, with participants being asked to choose from lists of responses.

We asked whether practitioners had been involved in the treatment; answers classified to 'no', 'yes throughout' or 'yes at some stage only'. Participants were also asked detailed questions, where applicable, about how they were introduced to the treatment; reasons for using it, and reasons for stopping it. Participants were also asked how helpful they had found the treatment, scored on a numeric visual analogue scale (VAS) ranging from (+10) 'very helpful' to (-10) 'very harmful', with zero defining 'not helpful and not harmful'. Current use of a treatment was defined as 'use during the week leading to the interview'. Those who had stopped a treatment were asked about the reasons for stopping. Finally participants were asked about their intention for future use of each current or previously used treatment. In the list of options for each answer, there was an 'other' option which allowed participants to freely state his or her answer if different from the listed ones.

At the end of the interview the Chronic Pain Grade (CPG) questionnaire [29] was self-completed by participants. This seven item self-complete instrument provides a score of severity, enabling chronic pain patients to be classified into one of four categories which combine persistence (duration), intensity and disability:- Grade I, low disability-low intensity; Grade II, low disability-high intensity; Grade III, high disability-moderately limiting; and Grade IV, high disability-severely limiting. Its use has been validated in the USA and the UK [30].

All interviews were conducted between April and July 2004 by one researcher (MA). The format was piloted with five patients from a general practice outside the study. 10 random interviews from the main study were videoed and studied by an independent qualitative researcher at our Centre, applying criteria for quality developed by De Vaus [31].

### CAM and conventional treatments

Our identification of treatments as 'CAM' and 'conventional' was based on our literature review, and on the views of 26 clinical and non-clinical researchers in our Centre, whose main topic of research is musculoskeletal pain. We surveyed the latter about how they would classify the list of treatments used in our main survey. Although this was not a formal Delphi procedure, the aim was to provide a common-sense, informed list to reflect current ideas about whether an individual therapy is more or less 'CAM'. 51 treatments were classified as 'CAM' and 23 as conventional (Table 1).

One of the difficulties facing research in the field of CAM is defining this type of 'medicine'. It is a heterogeneous group of therapies, substances, supplements, procedures, techniques, rituals, practices, systems etc, which people use, do and undergo while seeking to alleviate health problems or to maintain health. Because of their great diversity, the only way to identify these treatments as a distinct group seems to have been by 'negatively' defining them as treatments that are not taught in medical schools or provided in hospitals. In 1982, this group of treatments was defined as *"treatments that a conventional unit is unlikely to prescribe" *[14]. Within that were included aids for the home (used by patients with arthritis) as well as herbs and acupuncture. Other researchers defined CAM as a *"name given to a system of healthcare that lies predominantly outside the mainstream of conventional medicine" *[15].

Ernst [24] defined this type of medicine as *"diagnosis, treatment and/or prevention which complements mainstream medicine by contributing to a common whole, by satisfying a demand not met by orthodoxy or by diversifying the conceptual frameworks of medicine"*. A definition of CAM adopted by the Cochrane Collaboration is "*Complementary and alternative medicine (CAM) is a broad domain of healing resources that encompasses all health systems, modalities and practices and their accompanying theories and beliefs, other than those intrinsic to the politically dominant health system of a particular society or culture in a given historical period. CAM includes all such practices and ideas self-defined by their users as preventing or treating illness or promoting health and well-being. Boundaries within CAM and between the CAM domain and that of the dominant system are not always sharp or fixed*" [50]

Another definition which was coined by Eisenberg in 1993 in the USA and was then widely adopted is *"medical interventions not taught widely at U.S. schools or generally available at U.S. hospitals" *[8]. The World Health Organisation's definition of CAM is *"all forms of health care which usually lie outside the official health sector" *[54]. The BMA definition is that CAM covers *"forms of treatments not widely used by orthodox healthcare professions....skills of which are not taught as part of the undergraduate curriculum of orthodox medical and paramedical health care courses" *[54]

### Statistical analysis

Data was entered using Windows Excel 2000 and analysed using SPSS 12 for Windows. The dependent variable for statistical purposes was CAM use (yes/no). CAM use was further sub-classified as CAM use only; CAM use in addition to use of conventional treatment. Prevalence estimates including confidence intervals were calculated and statistical testing of differences in prevalence between subgroups was carried out using the chi-square test.

We investigated non-response to participate in our interviews by using data from the original health surveys undertaken by potential participants and compared those who agreed to interview with those who did not with respect to socio-demographic characteristics, general health status (as measured by the Short-Form 36), and lower limb joint pain and disability (as measured by the WOMAC questionnaire), as well as use of prescribed pain medication and home remedies for pain.

## Results

### Response and sample characteristics

Of the 427 patients invited, 138 participants responded and all of them attended and completed the interviews (response 32.3%). There were 39 (28%) in the age group 18–59 years, 51 (37%) between 60–69 years and 48 (35%) in the group 70 years or over; 55 participants were male (40%) and 83 female (60%). Distribution by occupational category was 68 (49%) professional or managerial, 29 (21%) intermediate, and 41 (30%) routine and/or manual. 37 participants reported pain in CPG grade I (27%); 46 (33%) in grade II; 32 (23%) in grade III; and 23 (17%) in grade IV. 'Lower back' and 'knee' were the most commonly reported areas of pain (52% and 48% respectively). The majority (77%) reported pain in more than one area.

### Non-response

There were insignificant differences between respondents and non-respondents in gender, age, health status or severity of pain and disability. No differences appeared either for health care use between the two groups (42% of those who used at least one prescribed medication responded, compared with 41% who did not; and 46% of those who used at least one 'home remedy' responded, compared with 39% who did not).

### Use of CAM and conventional treatments

116 interview participants (84%, 95% CI = 78% – 90%) said they had used at least one CAM treatment for pain in the previous year; most (75/116, 65%) being current users. The total number of CAM treatments ('episodes of treatment use') reported by all CAM users was 321, which represents an average of 2.8 episodes of CAM use in this subgroup. Our study population represented users of conventional services by virtue of all being GP consulters. Not all of them, however, had been actually using conventional treatment for their pain during the previous year, even though they had seen their GP for their pain during the same period. We therefore looked at the actual use of conventional treatment. A total of 111 interview participants (80%, 95% CI = 74% – 86%) reported using at least one conventional treatment, mainly prescribed medication. 21 participants (15%) had used CAM treatments only; 16 (12%) conventional treatments only; 95 (69%) had used both CAM and conventional treatments, and 6 (4%) had not used CAM or conventional treatments. Thus, most CAM users (95/116, 82%) and most conventional treatment users (95/111, 86%) had used both during the time period of recall.

Using the treatment list in the interviews led to an increase in reporting of treatment use. The total number of occasions on which any type of treatment (CAM and conventional) had been mentioned as used at least once by participants was 556, a mean of 4 treatments per person. For 224 of these occasions, treatments were reported with the aid of the list, representing an average increase of reporting, after the list was shown, of 1.7 treatments per person. The increased rate of reporting was higher for CAM treatments (increased by 51%) compared with conventional treatments (increased by 25.5%).

The prevalence of use of individual CAM treatments, expressed as the proportion of all interviewees who reported using a CAM treatment at least once, is shown in table 2. In total, 32 of the 52 CAM treatments included on the pre-specified lists had been used by at least one of the study participants, and 28 further treatment names had been used but not been included on the lists, 16 of which were CAM (table 1). These 16 CAM treatments were each used by either one or two participants, apart from exercises which were mentioned as used by 19 participants.

**Table 2:** Frequency of CAM treatment use, excluding 'other' CAM.

**Treatment**	**CAM users *(Total n 116)***	
	
	**n**	**%**
Glucosamine	44	38
Fish oil	41	35
Massage Therapy	16	14
Copper bracelets	15	13
Magnets	15	13
Electrotherapy	12	10
Chondroitin sulphate	10	9
Osteopathic manipulation	10	9
Acupuncture	9	8
Aromatherapy	9	8
Evening primrose	7	6
Weight loss program	5	4
Devil's claw	5	4
Relaxation	5	4
Pilates	4	3
Prayer	4	3
Lifestyle program	3	3
Ginger	3	3
Acupressure	3	3
Reflexology	3	3
Soft tissue mobilisation	3	3
Yoga	3	3
Homeopathy	3	3
Chiropractic	2	2
Feverfew extract	1	1
Turmeric	1	1
Myofascial release	1	1
Therapeutic touch	1	1
Guided Imagery	1	1
Pet Therapy	1	1
Energy Healing	1	1
Hydrotherapy	1	1

### Characteristics of treatment users

Table 3 compares CAM and conventional treatment use by age, gender, socio-economic classification and Chronic Pain Grade. There was an inverse association between age and CAM use – older patients were significantly less likely to use CAM for pain control. Men were little different from women in their overall use of CAM, though women were significantly more likely to use conventional treatments, and hence the combined use of CAM and conventional treatment was significantly higher in women.

**Table 3:** Use of CAM and conventional treatments by socio-demographiccharacteristics and severity of pain.

	**n**	**CAM treatment use n (%)**		**Conventional treatment use n (%)**		**Combined use n (%)**	
		
		**Yes**	**No**	**Yes**	**No**	**Yes**	**No**
All participants	138	116 (84)	22 (16)	111 (80)	27 (20)	95 (69)	43 (31)
*Age groups*							
18–59	39	37 (95)	2 (5)	32 (82)	7 (18)	31 (79)	8 (21)
60–69	51	44 (86)	7 (12)	42 (82)	9 (18)	36 (71)	15 (29)
70+	48	35 (73)	13 (27)	37 (77)	11 (23)	28 (58)	20 (42)
		p = 0.005*		p = 0.544		p = 0.033	
*Gender*							
Male	55	43 (78)	12 (22)	33 (60)	22 (40)	30 (55)	25 (45)
Female	83	73 (88)	10 (12)	78 (94)	5 (6)	65 (78)	18 (22)
		p = 0.125		p < 0.001		p = 0.003	
*Socio-economic class*							
Professional or managerial	68	52 (76)	16 (24)	50 (74)	18 (26)	42 (62)	26 (38)
Intermediate	29	29 (100)	0 (0)	26 (90)	3 (10)	24 (83)	5 (17)
Routine or manual	41	35 (85)	6 (15)	35 (85)	6 (15)	29 (71)	12 (29)
		p = 0.014		p = 0.119		p = 0.118	
*CPG*							
I	37	28 (76)	9 (24)	25 (68)	12 (32)	21 (57)	16 (43)
II	46	40 (87)	6 (13)	37 (80)	9 (20)	32 (70)	14 (30)
III	32	29 (91)	3 (9)	28 (88)	4 (12)	25 (78)	7 (22)
IV	23	19 (83)	4 (17)	21 (91)	2 (9)	16 (70)	7 (30)
		p = 0.310		p = 0.013		p = 0.156	

P-values were derived by chi square test (test for linear trend in the case of age group and CPG)

CAM use was significantly different between socio-economic classes; CAM use was higher in intermediate and routine and manual occupations than in professional and managerial occupations. A higher percentage of CAM use was reported in those with CPG of III-IV compared to those with CPG of I-II; the statistical test for trend was not significant however. There was a significant trend toward greater use of conventional treatments with increased CPG category: 91% of those with CPG-IV had used conventional treatments compare to 68% of patients with CPG-I.

### Reasons for using and stopping CAM treatments

Table 4 illustrates that the most commonly reported introduction to using at least one CAM treatment was through a recommendation from a friend or a relative (55/116, 47%). Most of the 116 CAM users (62, 53%) gave their reason for using at least one of their CAM treatments as: *'I like to try anything that may work'*. Of the 321 episodes in which CAM treatments were used, practitioners had been involved in 128 (40%). Forty-one CAM users (41, 35%) had stopped at least one of their CAM treatments. The total number of episodes in which CAM treatments were stopped was 144 (144/321, 45%). The reasons given for stopping CAM treatments are shown in table 4.

**Table 4:** Reasons for using and stopping CAM treatments.

**Ways participants introduced to CAM**	**n (%)**	**Reasons for using CAM**	**n (%)**	**Reasons for stopping CAM**	**n (%)**
Recommended by a friend or a relative.	55 (47)	I like to try anything that may work	62 (53)	I finished the treatment course	39 (27)
Prescribed, or referred to it, by a health professional	35 (30)	I was referred to it or it was prescribed for me	29 (25)	I don't think I need it anymore	13 (9)
Media (TV, radio, newspapers, Internet)	27 (23)	I find that it helps me in general not just for pain	23 (20)	Cannot afford it	6 (4)
Literature	10 (9)	I believe in it	22 (19)	It caused me problems or side effects	6 (4)
Practice it/involved with it	10 (9)	Other treatment caused me problems or side effects	2 (2)	I heard of a bad experience with it	1
				Not available where I live	0
				My doctor advised me against it	0
Other ways*	22 (19)	Other reasons*	19 (16)	Other reasons*	79 (55)
Found it in a shop		Brought in by a relative		Didn't help	40
Local advertisement		Compatible with birth sign		Only use it when I need it	31
Previous experience		Suggestion by others		Not practical	
Family experience		Persuaded by evidence		Caused more pain	
Experience at vet use		Natural product		Staining	
Workshop		Carried on, don't know			
Health farm		Family experience			
Own initiative		Has no side effects			
Practitioner is a friend		Previous experience			
The gym		Heard its good			
Social class		Recommended in a magazine			
		Thought it may work			
Total	116	Total	116	Total	144

*These other ways/reasons were not included in our lists and were offered freely by participants. Items are listed in a descending order according to how commonly they were mentioned.

### Perceived helpfulness of CAM

A summary of helpfulness scores given for the most commonly used CAM and conventional treatments is shown in figures 1a and 1b. Of the 14 most common CAM treatments, osteopathy, relaxation, aromatherapy and evening primrose had the highest median helpfulness scores i.e. 7 or above, whereas devil's claw and copper had the lowest median scores i.e. zero (Figure 1).

Harm (negative scores on a (-1) to (-10) VAS) was reported by eight users (8/116, 7%) from 7 CAM treatments. Harm scores ranged from the highest of -10 reported for acupuncture (one user) to -4 reported for acupupressure (one user), massage (2 users), electrotherapy (one user) and chiropractic (one user) to -3 for devil's claws (one user) and -2 for yoga (one user).

**Figure 2:**
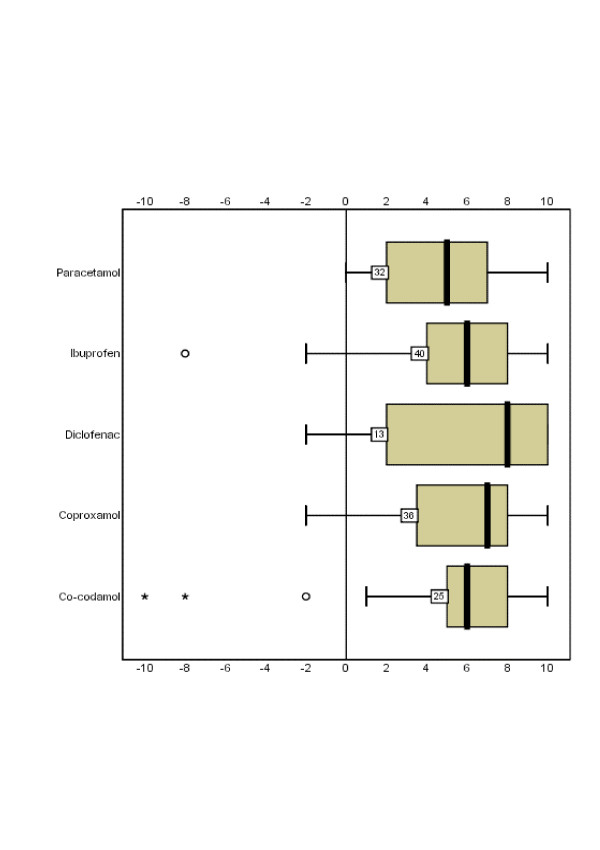
Boxplot summary of perceived helpfulness scores on the VAS for the most commonly used conventional treatments. Values represented are number of users and 5-number summary (median, 25th and 75th percentiles and range) and outlier observations (data that lie outside the interval: median ± 1.5× mid-spread).

All five of the most commonly used conventional treatments had median scores of 5 or above. However, not all responses to use of conventional treatments were positive (Figure 2).

### Intention on future use of CAM

Most CAM users said they would use the particular CAM treatment for pain in the future. Out of the 321 recorded uses of CAM treatment, users stated that they would use 277 (87%) of these again.

## Discussion

### Use of CAM and conventional treatment

Directly asking primary care consulters with chronic musculoskeletal pain about how they treated their pain, without indicating in the question that we were interested in any particular class or group or type of treatment and not asking the interviewees to distinguish between conventional or CAM therapy, we found the prevalence of CAM use high. More than four of every five patients interviewed had used at least one CAM treatment, and more than two thirds of CAM users were current users, representing more than half of the study sample.

Using an open question alone, even though clearly explaining that we were interested in every type of treatment used, would still have provided us with incomplete information, had we not used the list which appeared to lead participants to mention more treatments. This could be a simple issue of recall, but could also represent their reluctance to admit to using certain treatments, especially knowing that the interviewer was a doctor. Previous surveys have found a similar effect [23,24,31]. The prevalence of CAM use, in one survey, increased from 56% to 85% when introducing a treatment list [33].

It has been shown that the larger the number of treatments included on the list, the larger will be the estimates of prevalence.[6,34]. It is possible that using such lists could trigger incorrect memory or confuse participants into thinking that they have used treatments that they have not, because of similarities either in names or in actual treatment technique (e.g. acupuncture and acupressure). Such a possibility could inflate the prevalence with inaccurate estimates. This has led some researchers to confine their exploratory work to a small number of treatments in order to obtain precise information [6]. We were aware of this issue and the trade-off between obtaining accurate information on a small number of treatments and exploring all treatments that were being used for pain. Because we used face-to-face interviews, we used the opportunity to deal with any possible confusion and clarify that the participant had actually used the treatment. Such opportunity would not have been available if other methods had been used e.g. postal survey. Secondly, we asked a number of detailed questions about each treatment reported, and this would have reduced the chance that the participant might have mentioned the treatment by mistake.

The extent of the increase in reporting treatment use in conjunction with the list was twice as high with CAM as with conventional treatments (164/321, 51% *vs *60/235, 25.5%). A possible explanation is that participants might have been less likely to volunteer, to a doctor, information related to CAM compared with conventional treatments, an observation made in previous surveys [9,24,35]. This would correlate with the comments of many participants that they did not realise that we were also interested in these (CAM) treatments. We were not able to find a similar comparison between the effect of using a list on reporting CAM and conventional treatments among the published surveys in the literature.

We were not aware of published surveys that specifically targeted primary care patients in the UK to explore their CAM use for musculoskeletal pain. It is difficult therefore to compare our findings with those from previous surveys in the UK, many of which either targeted patients attending hospital clinics and who had known diagnoses e.g. rheumatoid arthritis [14,16,17,35,36], fibromyalgia [15,37], multiple sclerosis [38] or post-spinal cord injury [39] or targeted individuals in the community [10,11] suffering from chronic pain in general and not specifically musculoskeletal pain. Surveys that looked at CAM use in primary care in the UK mainly explored access to CAM and GPs' use and attitude towards it. It was found in a number of these surveys that between 39% and 83% of participating GPs were 'active' with respect to CAM, i.e. practising it, referring for it or endorsing it to their patients [4,5,19,20,22,23]. This could be one possible reason for the high rate of use of these treatments among their patients. We did not ask our participants whether some CAM treatments were practised by conventional health professionals. We know, however, that a third of CAM users in our study said that they came to use CAM because they were referred to it, or it was recommended, by a health professional. For a quarter of CAM users in our study, that was their main reason for using CAM treatments. This obviously refers only to CAM use for chronic musculoskeletal pain and does not include its use for other reasons. This could indicate high 'activity' in relation to CAM in the general practices in the area where we conducted our study, which could explain high CAM use among individuals like our sample of primary care patients. It would be interesting to explore any direct association between GPs activity with regard to CAM and its use among their patients.

In the USA, surveys have explored CAM use among primary care patients [40-42]. However, it was general use of CAM that was explored rather than use linked to a specific condition or symptom. In one survey [40], it was found that 21% of patients, interviewed while visiting their primary care doctor, had used CAM treatment for the medical problem linked with that GP visit. It is difficult to apply findings related to family medical practice in the USA with primary care in the UK because of the variations in structure, profile and activity.

Surveys among pain sufferers found the prevalence of CAM use ranging from 16% up to 100% in the UK [11,12,14-17] and the USA [10,35-39,43]. The variation in the prevalence figures is likely to reflect variation in survey methodologies. The majority of these surveys, however, showed the prevalence of CAM use to be consistently higher among pain sufferers compared with other patients.

The two main characteristics that our participants have, namely that they are actively using primary healthcare and that they suffer from chronic musculoskeletal pain, would make them, according to these previous surveys, the more likely users of CAM. This puts the high prevalence figure observed in our study into perspective.

Our study participants were, by selection, users of conventional healthcare, most having made more than two visits to their general practitioner in the previous year. We found that most of this study population were actually using conventional treatment in combination with CAM. This suggests that patients whom GPs saw most frequently for musculoskeletal pain were more likely than not to be using CAM treatments as well. This is consistent with the extensive use of CAM and conventional healthcare services by patients with chronic pain observed in previous surveys [11,44].

The high rate of combined use of CAM and conventional treatments could reflect high unmet needs. Surveys have shown that regardless of whether a chronic illness was reported, CAM users tend to report poorer health compared with non-users [40,44-46]. However, in one survey [46] it was found that use of CAM was more than twice as common among high users as among low users of medical services in general suggesting that high combined use represents a characteristic of the individuals and is unrelated to their health status or needs.

The high grade of severity of reported pain among our participants would probably be expected with our participants being active users of the health service because of their pain. The positive association between the frequency of GP visits and pain severity has been identified in previous surveys [11,47]. 60% of our participants had visited their GP at least twice during the previous year for musculoskeletal pain and 80% were using some form of conventional treatment, mainly prescribed medications. It appears, therefore, that the majority of our patients with chronic musculoskeletal pain who use both CAM and conventional treatments and are visiting their GP (for whatever reason) are still symptomatic and it is possible that this is one reason for their use of both types of treatment. This is important because it increases the relevance of the high use of CAM treatments by chronic musculoskeletal pain patients. It seems to tally with the common perception among GPs of the lack of effective treatments for such symptoms. This would lead some patients at least to try anything that might help with their pain.

It is important to note that by targeting chronic musculoskeletal pain sufferers who are using primary care services, a group of patients with the same pain and who are not using primary care services were not reached by this study. This is a potentially important group of patients, some of whom might be exclusively using CAM for pain. We cannot comment in this study on the use of CAM among such group and our findings remain only applicable to primary care consulters.

### CAM definition

The availability of a single agreed epidemiological definition is important for surveys if the results are to be comprehensible and comparable. The use of varied definitions for CAM, as was highlighted earlier, has its effect on research in this field. That effect is evident from the type of research questions used in these surveys and the wide range of prevalence figures for use of CAM. Considering the types of 'treatments' which fall under the umbrella of CAM, it seems that for a part of them at least local availability and recognition is important to correctly estimate the prevalence of their use. On the other hand, this local approach needs to be balanced by the need to accurately compare findings of various surveys.

We therefore have used a local consensus on what is considered as CAM or conventional treatment and we also present our findings for individual treatments to allow for differing definitions to be applied and for accurate comparison to be made.

### Socio-demographic characteristics of treatment users

The typical socio-demographic characteristics of the majority of CAM users in our study did not echo those from the majority of studies in which CAM users were found more likely to be women [11,48] from higher social class groupings. [9,11,44,46,48] There are, however, studies which did not find a link between higher CAM use and higher income [40] or any significant difference by gender among CAM users [5,35,48].

The reason for the contrast between our results and other studies' might lie in regional variations in CAM use [46,49] or might represent patterns specific to sufferers of chronic musculoskeletal pain.

It has been suggested that the observed regional variation in CAM use more likely to reflect variation in access and availability than regional differences in public attitude and interest [50]. Access to these treatments can be severely restricted, with 90% of CAM provided in the private sector [44], leading to the suggestion that its use is related to the affluence of the area [4]. Surveys have shown that CAM use in the south west of England, for example, was higher than the national average (16% *vs *10%) [22].

Geographical variation in the availability and provision of CAM has been suggested as another possible explanation for variation in use [4,11,50]. One factor that was shown to influence CAM availability is the nature of local conventional healthcare services and primary care in particular (i.e. practices' attitude towards CAM and its provision; GP's special interest in CAM or antipathy towards it) [22]. GP endorsement of these treatments varied between areas (38% in Liverpool area *vs *54% in the south west of England) [22,23] as well as their active involvement i.e. practicing CAM [4,22]. Variation in demand could also influence availability of CAM. It has been shown that the prevalence of chronic pain, one of the most common health problems for which CAM is used, varied widely across geographical areas. [51]

It is interesting to attempt to explain our finding of the higher use of CAM and conventional treatments combined among women compared with men. It has been shown in one survey at least, that women were more likely than men to report chronic pain with no difference between genders in the reported severity of pain.[47,52] which could arguably offer an explanation. Women were also more likely to report high expressed needs than men [47]. In another study, where use of healthcare services was explored, women were found more likely to have used prescription and non-prescription medications, alternative therapist and alternative medication [11]. This could suggest that the high use among women, compared with men, of both conventional and unconventional medicine for pain, is related to their higher expressed needs and not to the severity of the reported pain.

### Perceived helpfulness from using CAM

Attempts to assess this have been made in past surveys from information mainly based on doctors' reports of their patients' benefit from using CAM [23,48,19,53]. However doctors' knowledge of their patients' use of CAM is often very limited [9,24] and the views of doctors and patients on the usefulness of CAM may differ [40].

There has been much recent debate about the lack of available evidence regarding the efficacy of CAM treatments. CAM treatments in our study were generally found to be helpful by participants, echoing previous findings from one systematic review [24]. This might represent what is called as the effectiveness gap [56], although in a reversed way. The effectiveness gap is said to exist when a treatment is shown to have an effect based on its pharmacological action but shows a smaller effectiveness in clinical practice. Here, the gap seems to exist when treatments (such as some CAM treatments) are perceived to be helpful by users when no evidence for their effect exists.

Although the number of participants who reported experiencing harm in the form of worsening pain symptoms following the use of some CAM treatments was small and although these data do not represent an objective measure of effectiveness, one conclusion is that, although beneficial effects on pain from each CAM treatment are commonly reported, many users do not perceive CAM to be automatically beneficial, and a number of them (substantial if extrapolated nationally) considered themselves to have experienced harmful effects.

The range of scores for perceived helpfulness from the commonly used conventional treatments was wider than for CAM treatments, and there were higher harm scores. The latter might be balanced or off-set by evidence of effectiveness the likes of which is lacking for many CAM treatments. Interestingly, some of the favourable CAM treatments, such as chondroitin sulphate and osteopathy, had higher average ratings for perceived helpfulness than paracetamol, ibuprofen and co-codamol. The differences in the numbers of users, however, make accurate comparison difficult beyond mathematical extrapolation. This issue merits further investigation.

An important finding in our study was the instances where participants reported harm attributed to the use of treatments. The eight instances of harm attributed to the use of seven CAM treatments represent a small percentage of the total number of instances on which CAM treatments were used. These seven CAM treatments had been used 52 times in this study's population.

Some observations could be made on these harm reports. Firstly, these harm scores were reported for some treatments that also received high perceived helpfulness scores from other users. Electrotherapy received nine positive scores (+2 to +8), massage received 14 positive scores (+1 to +10) and acupuncture received 5 positive scores (+3 to +9). Secondly, although the question was about perceived helpfulness in relation to pain, we believe that reported negative scores might not have always meant "worsening of pain following using the treatment" but might also meant other adverse effects which may not be related to pain. We did not expand on the nature of the harmful effect that was reported and this information was collected as a score on the negative arm of (-10) to (+10) VAS. Thirdly we do not know whether these effects were reliably caused by these treatments. The answers were purely subjective.

It is interesting to compare the number of these reported harmful incidents with the number of participants who said that they have stopped CAM because it caused them problems or side effects. These reasons for stopping were given on fewer occasions of CAM use (six) than reported harmful events (eight) and not all these cases are the same. This could either reflect the unreliability of the assessment made by the participants, or that some of them did report harm which was not of a type or severity that had made them stop the treatment. Finally, it seems that reporting harm was more likely to be related to practitioner dependent treatments, although the number of instances was too small to validate this conclusion.

Although the number of reported perceived harm instances is small, they are nevertheless important. They highlight the fact that these treatments are not universally experienced or perceived as harmless. They are also important in the debate about the safety of CAM and its integration within the mainstream health services.

Harmful events attributed to CAM use have been reported previously. 38% of GPs in one survey reported adverse effects related to CAM use by their patients [22]. In another survey, 21% of responding GPs reported similar harmful effects [23]. A survey in Australia found that 25% of users of naturopathy reported effects [55]. The adverse events reported in the surveys studied in a systematic review of the use of CAM in rheumatology were low [26]. In addition to users' views and perceptions on harm, doctors, on the other hand, have a different view of the harm they perceive and attribute to using CAM. In one survey 62% of the participating physicians suggested that CAM use prevents patients from getting proper treatment [21].

The issue of perceived helpfulness is important, with implications for safety, integration and future research. With the increasing use of CAM and the increasing amount of anecdotal evidence for its helpfulness, or otherwise, by users, some are suggesting that there should be room for debate as to who decides what is and what is not effective and on what basis, at least in the NHS [12].

### Future intention on using CAM

We could not find published surveys in the UK that addressed this issue, although it has been reported in American surveys [48] where strong intentions to use CAM again in the future were identified. Future use of CAM may be influenced by perceived helpfulness [48]. In our study, there was a contrast between the intention on future use of conventional treatments, which most participants felt would be strongly influenced by doctors' advice, and future use of CAM which appears to be more dependent on a wish to try anything that might help.

### Response and generalisability

A limitation to the study was the higher than anticipated reluctance to be interviewed, which meant that we had a final study population of 138 as opposed to the 180 pre-specified in the sample size pre-requisite. We had also underestimated the amount of CAM use. Revisiting the power calculation *post hoc *and taking a 20% difference in CAM use based on a greater base value of 80% CAM use in the study population, meant that given a sample size of 138 we had 79% power of detecting this difference if it existed i.e. there was little loss in power compared to the prior calculation.

We took advantage of the fact that our patients had completed earlier postal surveys to compare responders and non-responders with respect to gender, age, pain and health status scores and health care use in general, as well as use of specific 'home remedies' (e.g. cod liver oil) which had been enquired about in the postal questionnaires. Differences were small and it is unlikely that those interviewed represent an unusual sample of our target population with respect to their general experience of pain and willingness to use a variety of treatments. Furthermore CAM was not mentioned or referred to during our study, and so responders are unlikely to represent a group specifically interested in this topic.

Generalising our study findings to the wider population of all patients with chronic musculoskeletal pain who are using primary healthcare services in the UK would require caution. CAM use varies between different parts of the country [23,19], and this may influence use among consulters also. This variation might explain why the use of some individual CAM treatments, such as Homeopathy, was lower among our participants compared with other surveys' [6]. However it seems unlikely that the broad patterns identified here would differ substantially in other primary care settings.

## Conclusion

We have estimated the prevalence of CAM use among musculoskeletal pain consulters in primary care in the UK, by directly asking a sample of such patients about all the methods which they used to alleviate their pain. The high rate of CAM use and wide range of experience of benefit and harm strengthen the argument for research to quantify benefit and assess safety of this type of treatment. The fact that the majority of CAM users in our study remained active users of conventional medicine and that their use of CAM was related to the persistence of their pain further highlights the importance of the research on the optimal management of pain in primary care.

## Competing interests

The authors declare that they have no competing interests.

## Authors' contributions

Majid Artus proposed the idea of the survey, conducted the preparatory literature review, developed the interview questionnaire and conducted the interviews. Data was entered and checked by members of the Centre's research team. Analysis was conducted by Martyn Lewis and Majid Artus. The paper was written by Majid Artus, with contributions from Peter Croft and Martyn Lewis. The study, throughout all its stages, was supervised by Peter Croft.

## Pre-publication history

The pre-publication history for this paper can be accessed here:


